# Supplementary material for: Integrative transcriptome-wide analysis of atopic dermatitis for drug repositioning
Source: Commun Biol. 2022 Jun 22;5:615. doi: 10.1038/s42003-022-03564-w (PMC9213508; doi:10.1038/s42003-022-03564-w)
Supplement: Supplementary file 1 — Supplementary Information [file 42003_2022_3564_MOESM1_ESM.pdf]

Supplementary Table S1. Summary statistics of TWAS genes in transcriptome meta-analysis

Entrez ID	logFC	AveExpr	t	P.Value	adj.P.Val	B	Symbol
10139	0.336965	5.802987578	13.23945358	1.26E-33	9.46E-33	65.34964874	*ARFRP1*
11127	-0.181066361	5.753189821	-6.512492748	2.14E-10	4.77E-10	12.40999226	*KIF3A*
140685	0.261669318	4.327486221	7.796964413	5.16E-14	1.41E-13	20.58972141	*ZBTB46*
2312	-1.25099819	12.15579639	-12.24032791	1.25E-29	7.66E-29	56.19943554	*FLG*
339400	-0.307954752	3.639348256	-7.386307279	8.35E-13	2.13E-12	17.85090328	*FLG-AS1*
3570	0.361658157	6.585455114	10.07249762	1.70E-21	6.90E-21	37.61498637	*IL6R*
49860	-0.439538649	4.573153669	-7.905802617	2.42E-14	6.72E-14	21.33416371	*CRNN*
5017	0.272417586	7.289604587	4.673288566	4.01E-06	6.77E-06	2.854611467	*OVOL1*
50861	-0.137945345	5.576030784	-4.221722075	2.98E-05	4.74E-05	0.936519237	*STMN3*
55012	0.358621231	4.959704334	12.44091841	2.03E-30	1.30E-29	58.01085325	*PPP2R3C*
56731	-0.237037137	6.613052952	-9.038342413	6.10E-18	2.04E-17	29.51450414	*SLC2A4RG*
5993	0.328050784	5.653336207	13.34244486	4.80E-34	3.66E-33	66.31001993	*RFX5*
84619	0.259418292	5.967408782	8.916409383	1.54E-17	5.05E-17	28.5973985	*ZGPAT*
84991	0.022727315	6.457766906	1.203133492	0.229610126	0.258057971	7.072146333	*RBM17*
8974	-0.166410037	5.716092658	-7.713917009	9.14E-14	2.46E-13	20.02685513	*P4HA2*
9173	0.408097885	4.350817916	8.289200411	1.60E-15	4.71E-15	24.01649519	*IL1RL1*

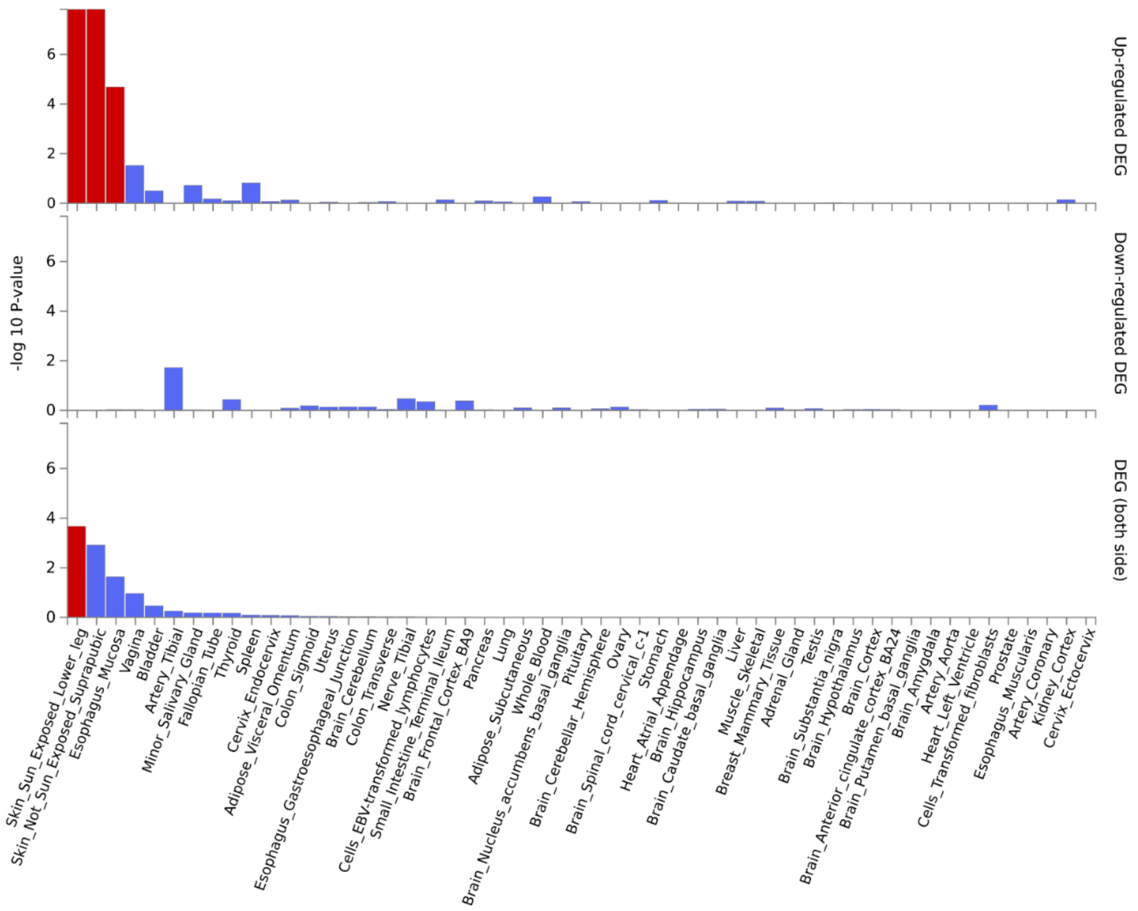

Supplementary Figure S1. Tissue type enrichment analysis of GWAS signals using FUMA

The bar plots of the gene-based tissue type enrichment analysis results using AD GWAS summary data using the GENE2FUNC process in FUMA web server. Upper, middle, and lower panel shows the enrichment of up-, down-regulated or both sides of DEGs in certain tissue denoted in x-axis, compared with other tissues, respectively. The height of the bars corresponds to the $-\log_{10}$(P-value) of the tissue type enrichment. Red bars indicate the significant enrichment of the tissue-specific expressions (Bonferroni corrected $P < 0.05$).

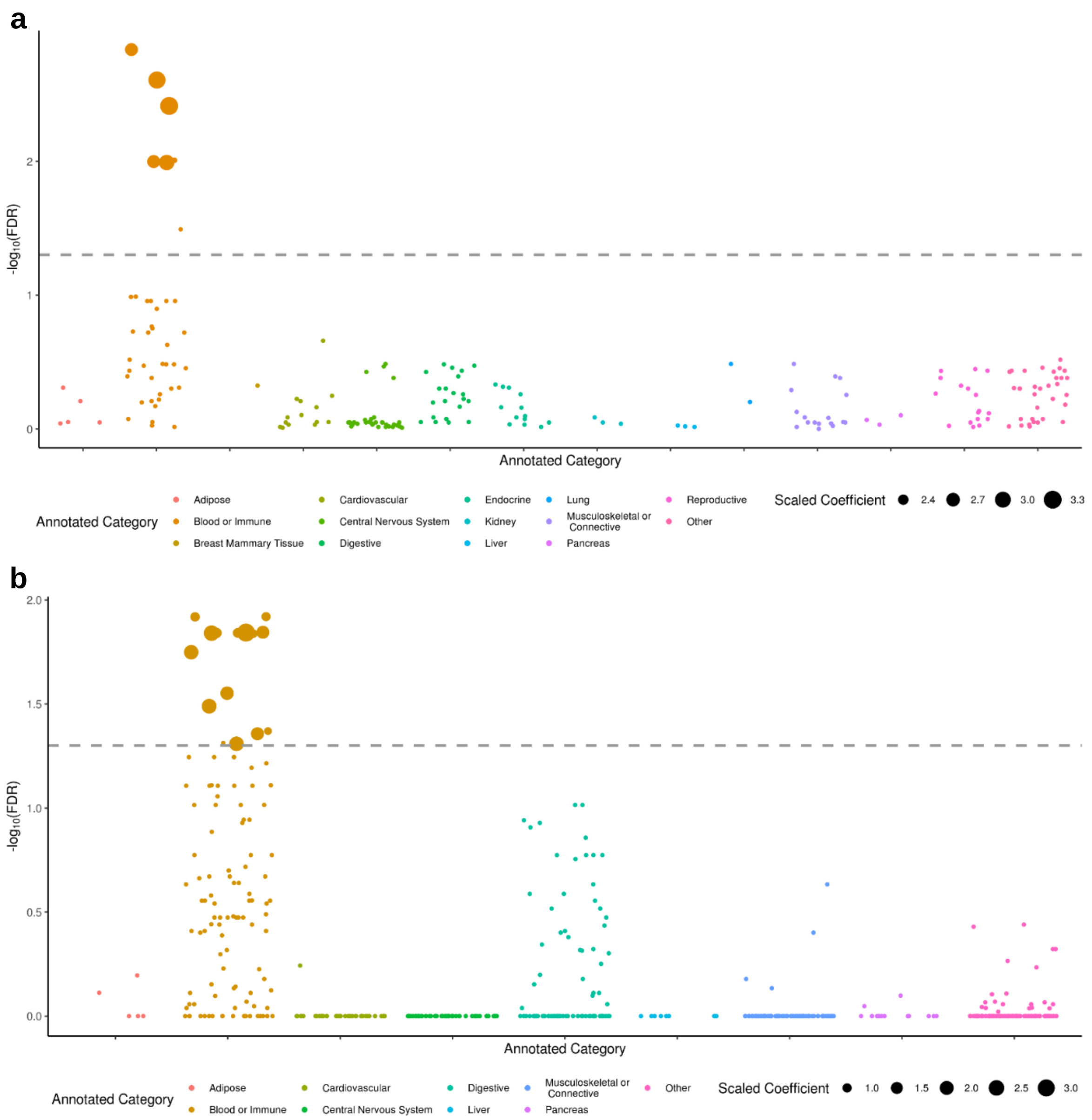

Supplementary Figure S2. Tissue or cell-specific heritability enrichment analysis of AD using LDSC-SEG

(a) A scatter plot showing the result of heritability enrichment analysis of AD GWAS summary statistics using the multi-tissue expression dataset. (b) A scatter plot of heritability enrichment results using the chromatin interaction data from multiple tissues or cell types. The color of the dots indicates the annotated category of the tissue or cell type as listed below each figure. For significantly enriched signals, the size of the dots are proportional to the scaled coefficient values. Grey line indicates the significance threshold of heritability enrichment (FDR < 0.05). Full table of the enrichment profiles is provided as Supplementary Table S1.

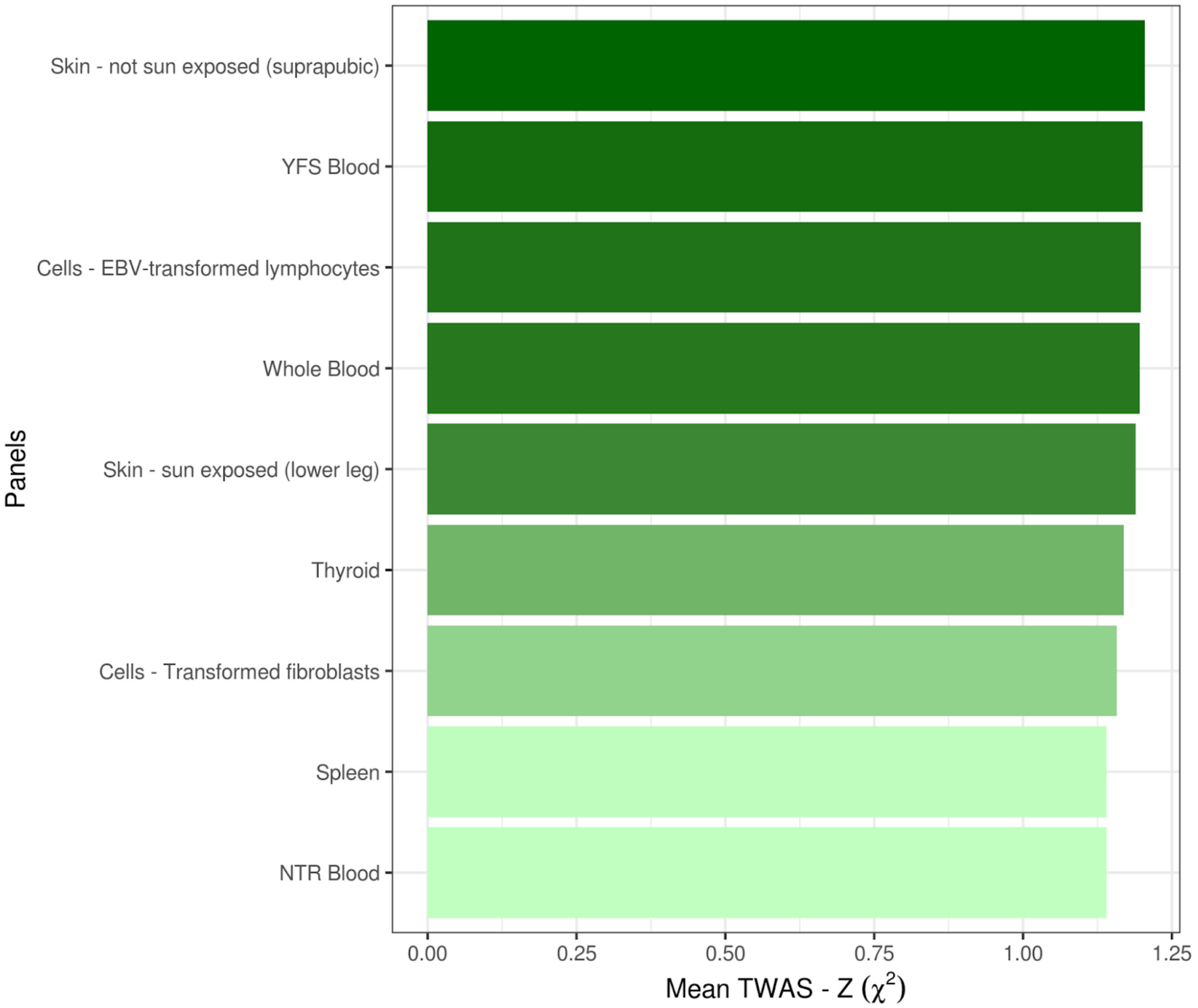

Supplementary Figure S3. Tissue-specific effect size of the TWAS signals
A bar plot showing the tissue-specific effects of TWAS signals. The x-axis and the shade of green of the bar indicate the mean of squared TWAS-Z scores that represents the effect size from overall TWAS signals in the corresponding tissue panel.

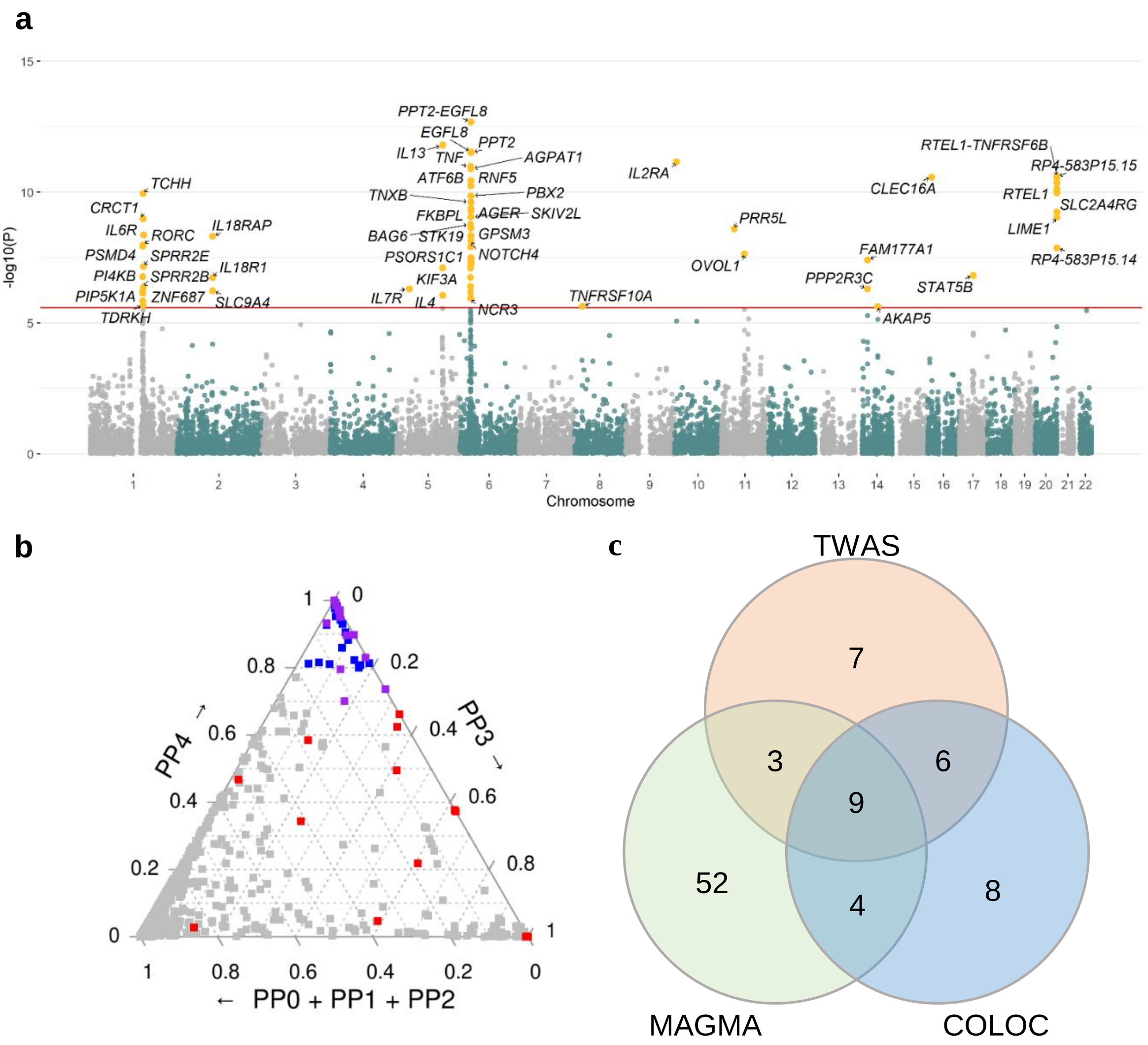

Supplementary Figure S4. Comparison of TWAS and other gene-prioritization methods

(a) A Manhattan plot showing the position-based gene prioritization results conducted using the MAGMA. The red line indicates the Bonferroni significant threshold ($P < 2.64 \times 10^{-6}$) and the yellow dots correspond to the 68 significant genes. (b) A ternary plot showing the result of COLOC analysis. Grey, blue, red, and purple points indicate the non-significant genes in any analyses, significantly colocalized genes in COLOC, significantly associated genes in TWAS, and significant genes in both COLOC and TWAS, respectively. (c) A Venn-diagram comparing the significantly prioritized genes from TWAS, MAGMA, and COLOC.

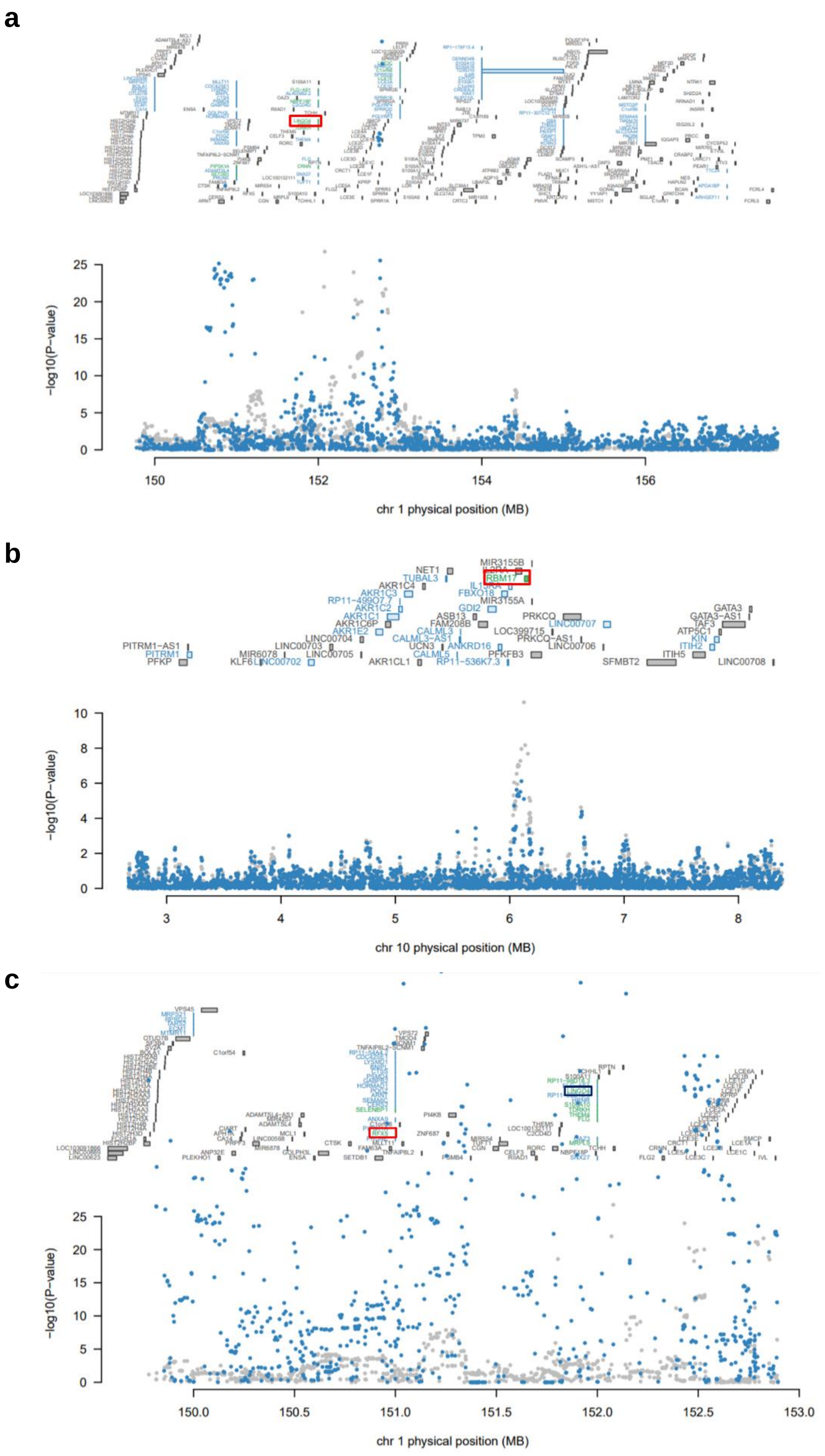

Supplementary Figure S5. Results of conditional and joint analysis of novel TWAS genes. Only jointly significant genes were displayed.

Regional association plots showing the association of (a) *LINGO4* in skin - not sun exposed tissue (chromosome 1), (b) *RBM17* in skin – sun exposed tissue (chromosome 10), and (c) *RFX5* in thyroid panel (chromosome 1). Jointly, marginally, and not significantly associated genes were displayed with green, blue and grey fonts, respectively. GWAS signals before and after conditioning on the expression of green gene were shown as grey and blue dots, respectively.

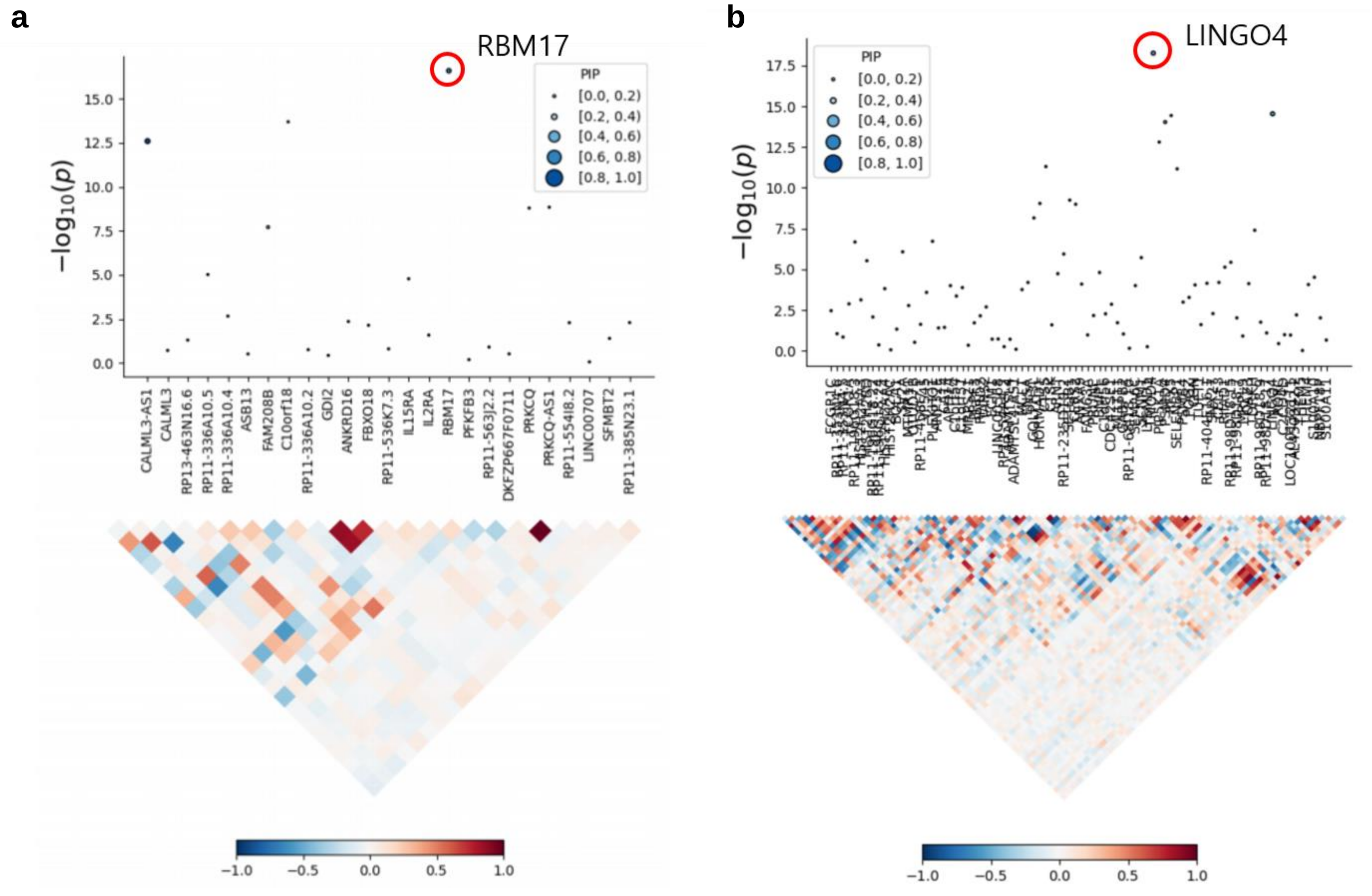

Supplementary Figure S6. Fine-mapping results of jointly significant novel TWAS genes using FOCUS. Only significantly prioritized genes were displayed.

Regional association plots of (a) *RBM17* in skin panel (chromosome 10) and (b) *LINGO4* in skin panel (chromosome 1). Y axis indicate the –log10(P-value) from FOCUS while x-axis represents the genes in the locus. Size of the dots are proportional to the PIP calculated with FOCUS. The correlation matrices below the association plots corresponds to the local correlation structure.

a b

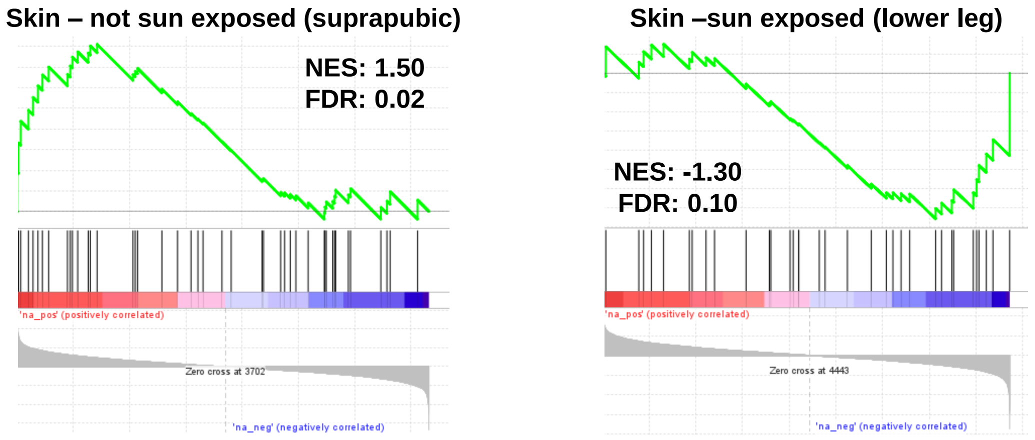

Supplementary Figure S7. Gene set level correlation analysis of TWAS and meta-analysis using GSEA

(a) A GSEA enrichment plot for TWAS signals from skin – not sun exposed (suprapubic) panel compared with up-regulated meta-signatures. (b) A GSEA enrichment plot comparing TWAS signals from skin – sun exposed (lower leg) panel and down-regulated meta-signatures.

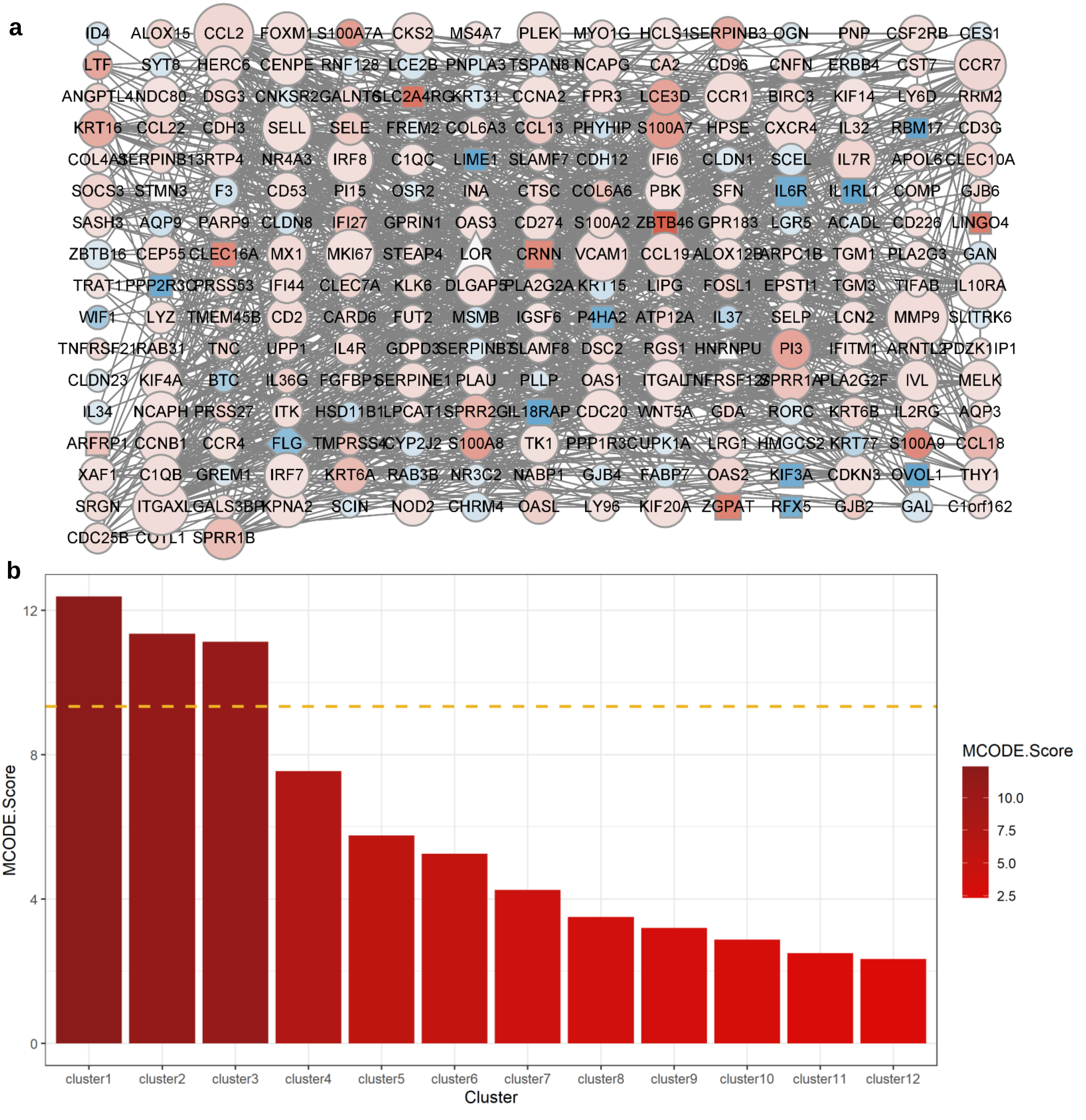

Supplementary Figure S8. Overall PPI-network constructed with TWAS genes and meta-signatures. (a) PPI-networks constructed using the STRING database with TWAS genes and meta-signatures. The size of the nodes are proportional to their degrees. Color of the nodes represents whether the gene was up- (red) or down-regulated (blue). Rectangle, circle and rhombi represents to the source of the gene, TWAS, meta-analysis, and STRING database, respectively. (b) Result of clustering analysis using MCODE application in Cytoscape. Color of the bar indicates the score calculated with MCODE. Yellow dashed line indicates the first quantile of the MCODE score.

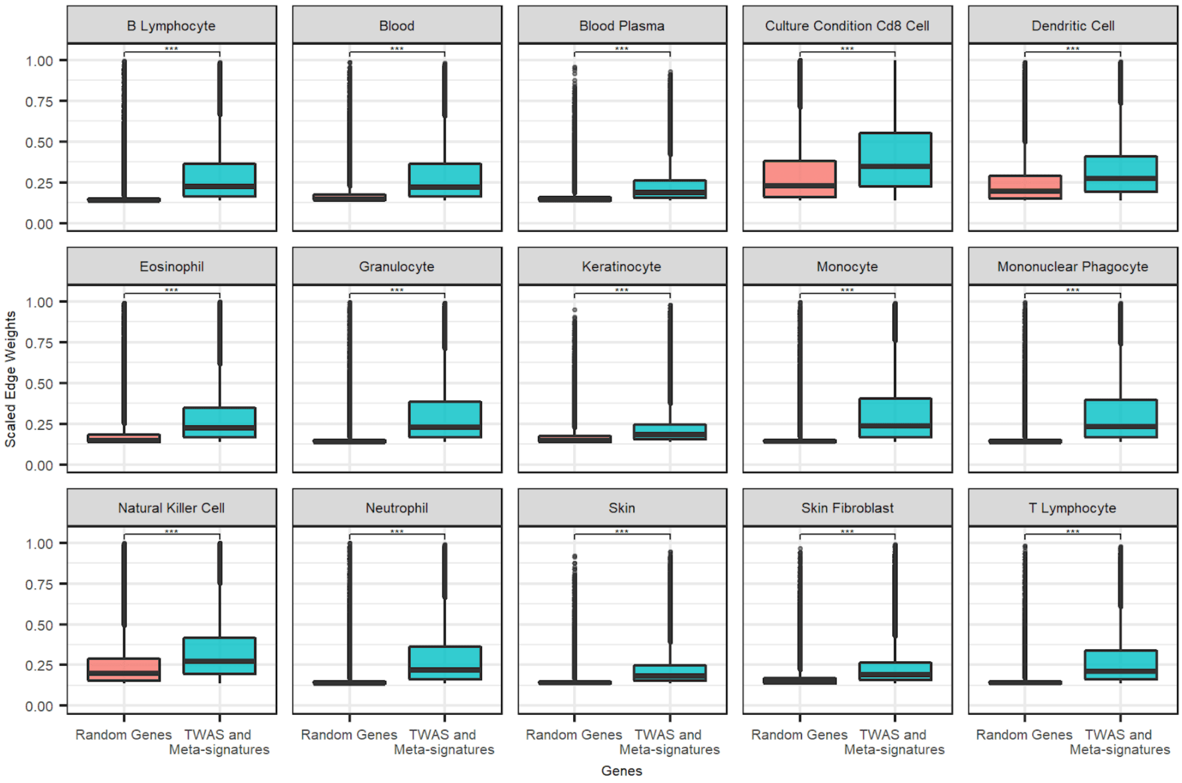

Supplementary Figure S9. The results of tissue- or cell-specific gene-gene connectivity analysis. The box plots of the tissue- or cell-specific analysis results. Gene-gene connectivity score between randomly selected genes and known AD markers were compared with the connectivity score between genes from TWAS and/or meta-analysis and known AD genes. Red and blue boxes indicate the rescaled connectivity scores from the randomly selected genes and TWAS genes and/or meta-signatures, respectively. *** P < 0.001
