# Supplementary material for: Integrative transcriptome-wide analysis of atopic dermatitis for drug repositioning
Source: Commun Biol. 2022 Jun 22;5:615. doi: 10.1038/s42003-022-03564-w (PMC9213508; doi:10.1038/s42003-022-03564-w)
Supplement: Supplementary file 3 — Reporting Summary [file 42003_2022_3564_MOESM3_ESM.pdf]

nature portfolio

Corresponding author(s): Wonhee Jang

Last updated by author(s): May 17, 2022

Reporting Summary

Nature Portfolio wishes to improve the reproducibility of the work that we publish. This form provides structure for consistency and transparency in reporting. For further information on Nature Portfolio policies, see our Editorial Policies and the Editorial Policy Checklist.

Statistics

For all statistical analyses, confirm that the following items are present in the figure legend, table legend, main text, or Methods section.

n/a	Confirmed	
☐	☒	The exact sample size (*n*) for each experimental group/condition, given as a discrete number and unit of measurement
☐	☒	A statement on whether measurements were taken from distinct samples or whether the same sample was measured repeatedly
☐	☒	The statistical test(s) used AND whether they are one- or two-sided<br>*Only common tests should be described solely by name; describe more complex techniques in the Methods section.*
☐	☒	A description of all covariates tested
☐	☒	A description of any assumptions or corrections, such as tests of normality and adjustment for multiple comparisons
☐	☒	A full description of the statistical parameters including central tendency (e.g. means) or other basic estimates (e.g. regression coefficient) AND variation (e.g. standard deviation) or associated estimates of uncertainty (e.g. confidence intervals)
☐	☒	For null hypothesis testing, the test statistic (e.g. *F*, *t*, *r*) with confidence intervals, effect sizes, degrees of freedom and *P* value noted<br>*Give P values as exact values whenever suitable.*
☒	☐	For Bayesian analysis, information on the choice of priors and Markov chain Monte Carlo settings
☐	☒	For hierarchical and complex designs, identification of the appropriate level for tests and full reporting of outcomes
☐	☒	Estimates of effect sizes (e.g. Cohen's *d*, Pearson's *r*), indicating how they were calculated

Our web collection on statistics for biologists contains articles on many of the points above.

Software and code

Policy information about availability of computer code

Data collection	The GWAS summary statistics used in this study can be found in GWAS Atlas (https://atlas.ctglab.nl/) with the accession ID 3606. Multi-tissue expression or chromatin datasets for LDSC-SEG analysis can be found in following github page (https://github.com/bulik/ldsc/wiki/Cell-type-specific-analyses). Tissue-specific eQTL panels can be found in GTEx Portal (https://gtexportal.org/home/), and pre-computed weights can be downloaded from the FUSION web page (http://gusevlab.org/projects/fusion/). Transcriptome data from AD patients are available in NCBI-GEO (GSE121212, GSE16161, GSE5667, and GSE120721) and EBI-ArrayExpress (E-MTAB-8149). Previously reported AD marker genes were searched on Open Targets Platform (https://platform.opentargets.org/). Tissue- and cell type-specific reference networks were retrieved from HumanBase (https://hb.flatironinstitute.org/). Functional gene sets retrieved from MsigDB (http://software.broadinstitute.org/gsea/msigdb) were used in this study.
Data analysis	The following tools, software, and packages were used in this study:<br>FUMA: https://fuma.ctglab.nl/;<br>FUSION: http://gusevlab.org/projects/fusion/;<br>LDSC, version 1.0.1: https://github.com/bulik/ldsc;<br>FOCUS, version 0.6.10: https://github.com/bogdanlab/focus;<br>TWAS-GSEA, version 1.2: https://github.com/opain/TWAS-GSEA;<br>sva, version 3.34.0: https://www.bioconductor.org /packages/release/bioc/html/sva.html;<br>limma, version 3.42.2: https://www.bioconductor.org/ packages/release/bioc/html/limma.html;<br>oligo, version 1.54.1: https://www.bioconductor.org/ packages/release/bioc/html/oligo.html;<br>edgeR, version 3.32.1: https://www.bioconductor.org/ packages/release/bioc/html/edgeR.html;<br>DESeq2, version 1.26.0: https://www. bioconductor.org/packages/release/bioc/html/DESeq2.html;<br>GSEA, version 4.1.0: https://www.gsea-msigdb.org/gsea/index.jsp;<br>STRING: https://string-db.org/;<br>Cytoscape, version 3.8.2: https://cytoscape.org/;

HumanBase, https://hb.flatironinstitute.org/;
CMAP, https://portals. broadinstitute.org/cmap/;
MANTRA 2.0: https://mantra.tigem.it/;
Rcpi, version 1.26.0: https://www.bioconductor.org/packages/release/bioc/html/Rcpi.html;
rcdk, version 3.5.0: https://cran.r-project.org/web/packages/rcdk/index.html;
and ggrepel, version 0.8.2: https://cran.r-project.org/web/packages/ggrepel/index.html.

For manuscripts utilizing custom algorithms or software that are central to the research but not yet described in published literature, software must be made available to editors and reviewers. We strongly encourage code deposition in a community repository (e.g. GitHub). See the Nature Portfolio guidelines for submitting code & software for further information.

Data

Policy information about availability of data

All manuscripts must include a data availability statement. This statement should provide the following information, where applicable:

- Accession codes, unique identifiers, or web links for publicly available datasets
- A description of any restrictions on data availability
- For clinical datasets or third party data, please ensure that the statement adheres to our policy

Overall TWAS results are provided as supplementary material. The results from other analysis are available from the corresponding author upon reasonable request.

Field-specific reporting

Please select the one below that is the best fit for your research. If you are not sure, read the appropriate sections before making your selection.

☒ Life sciences ☐ Behavioural & social sciences ☐ Ecological, evolutionary & environmental sciences

For a reference copy of the document with all sections, see nature.com/documents/nr-reporting-summary-flat.pdf

Life sciences study design

All studies must disclose on these points even when the disclosure is negative.

Sample size	GWAS data (control: 279,476; AD: 9,831; total: 289,307)<br>GSE121212 (control: 38; AD: 27; total: 65)<br>GSE16161 (control: 9; AD: 9; total: 18)<br>GSE5667 (control:5; AD:6; total: 11)<br>GSE120721 (control: 22; AD: 15; total: 37)<br>E-MTAB-8149 (control: 19; AD 83; total: 102)
Data exclusions	In transcriptome meta-analysis, samples obtained from healthy controls or AD patients were used for analysis.<br>Other conditions such as psoriasis were excluded from original dataset.
Replication	Unfortunately, because we used the currently largest AD GWAS data and there were no other AD GWAS data from larger European cohort, we were unable to replicate signals by utilizing independent GWAS datasets.<br>Instead, we conducted permutation analysis in order to provide the robustness of our results.<br>Additionally, we searched for previous studies to validate our TWAS results and confirmed that majority of our findings were well conformed with previous findings.<br>To rigorously assess our novel findings from TWAS that were not mentioned in previous studies, we validated those genes with 2 additional processes: conditional/joint analysis and fine-mapping.
Randomization	Not relevant for this study.
Blinding	Not relevant for this study.

Reporting for specific materials, systems and methods

We require information from authors about some types of materials, experimental systems and methods used in many studies. Here, indicate whether each material, system or method listed is relevant to your study. If you are not sure if a list item applies to your research, read the appropriate section before selecting a response.

Materials & experimental systems

n/a	Involved in the study
☒	☐ Antibodies
☒	☐ Eukaryotic cell lines
☒	☐ Palaeontology and archaeology
☒	☐ Animals and other organisms
☐	☒ Human research participants
☒	☐ Clinical data
☒	☐ Dual use research of concern

Methods

n/a	Involved in the study
☒	☐ ChIP-seq
☒	☐ Flow cytometry
☒	☐ MRI-based neuroimaging

Human research participants

Policy information about studies involving human research participants

Population characteristics	Participant characteristics of GWAS data are available in Watanabe et al., Nat. Genet. (2019) with PMID 31427789. Sample information and descriptions can be found on GEO or ArrayExpress with following accession IDs: GSE121212 (PMID: 30641038), GSE16161 (PMID 20004782), GSE5667 (PMID 17181634 and PMID 20625511), GSE120721 (PMID 25567045), and E-MTAB-8149 (PMID 31619666).
Recruitment	Recruitment information can be found on abovementioned articles and databases.
Ethics oversight	Ethics as reported by original publications below.<br>GWAS data: PMID 31427789<br>GSE121212: PMID 30641038<br>GSE16161: PMID 20004782<br>GSE5667: PMID 17181634 and PMID 20625511<br>GSE120721: 25567045<br>E-MTAB-8149: PMID 31619666

Note that full information on the approval of the study protocol must also be provided in the manuscript.
