# Supplementary material for: Integrative transcriptome-wide analysis of atopic dermatitis for drug repositioning
Source: Commun Biol. 2022 Jun 22;5:615. doi: 10.1038/s42003-022-03564-w (PMC9213508; doi:10.1038/s42003-022-03564-w)
Supplement: Supplementary file 5 — Description of Additional Supplementary Files [file 42003_2022_3564_MOESM5_ESM.pdf]

Description of Additional Supplementary Files

File name: Supplementary Data 1

Description: Results of tissue and cell-specific heritability enrichment analysis of AD using LDSC-SEG with multi-tissue expression dataset.

File name: Supplementary Data 2

Description: Results of tissue and cell-specific heritability enrichment analysis of AD using LDSC-SEG with multi-tissue chromatin dataset.

File name: Supplementary Data 3

Description: All results from TWAS.

File name: Supplementary Data 4

Description: List of every gene set that was significantly enriched in at least one tissue panel.

File name: Supplementary Data 5

Description: Overall GSEA results of meta-signatures.

File name: Supplementary Data 6

Description: Enrichment score and product score for each perturbation
